# Celebrating 20 Years of Open Access and Innovation at JMIR Publications

**DOI:** 10.2196/17578

**Published:** 2019-12-23

**Authors:** Gunther Eysenbach

**Affiliations:** 1 JMIR Publications Toronto, ON Canada

**Keywords:** JMIR, internet, medical informatics, ehealth, digital health, participatory medicine, open access, electronic publishing, scholarly publishing, science communication, journalogy, history of science, overlay journal, preprints, open science

## Abstract

In this 20th anniversary theme issue, we are celebrating how JMIR Publications, an innovative publisher deeply rooted in academia and created by scientists for scientists, pioneered the open access model, is advancing digital health research, is disrupting the scholarly publishing world, and is helping to empower patients. All this has been made possible by the disintermediating power of the internet. And we are not done innovating: Our new series of “superjournals,” called JMIRx, will provide a glimpse into what we see as the future and end goal in scholarly publishing: open science. In this model, the vast majority of papers will be published on preprint servers first, with “overlay” journals then competing to peer review and publish peer-reviewed “versions of record” of the best papers.

## Twenty Years Ago...

Twenty years ago, JMIR (originally the *Journal of Medical Internet Research*, the journal; now, JMIR Publications, the publisher) started an unprecedented journey to disrupt the medical informatics and publishing world with a new kind of journal and a new type of business model. The term “open access” was not invented back in 1999; most journals were still published and distributed on paper, and I remember the days of having to courier 10 copies of a manuscript on paper to a journal office, which then sent out the same hardcopies by postal mail to peer-reviewers. Creative Commons was in its infancy, and authors routinely signed away their copyright to publishers. A paper I submitted to a medical informatics journal took 2 years to be published. In this environment I was excited about the possibilities of the internet but also frustrated by the obvious inefficiencies and anachronisms of how we communicate in science. Having already worked as editor and book author in my student days, I decided to create a radically different, new journal for the internet age, with the intention of innovating on *form*, *content,* and *business model*.

In terms of *form*, the journal was free, rapid, and online only, and communication with authors and reviewers was exclusively electronic (we take that for granted now, but back then, most communication was still done via mail). This new form also opened up content to nontraditional audiences, ultimately contributing to a shift in the power distribution between doctors versus patients [1].

In terms of *content*, I solicited and encouraged original research focusing on “eHealth” (electronic health) [2,3] (called “digital health” by some today), which is a much broader concept than the “electronic medical record,” "decision support for clinicians", or "hospital information systems"—topics that dominated and defined the original discipline of medical informatics then (and partly still do now). I clearly saw already back then that information and communication technologies related to the internet would have an impact far beyond billing and record management [2,3].

In terms of the *business model*, my original idea was something like “we’ll figure it out, but it shouldn’t be subscription,” which only later morphed into “author/funder pays.” As a side note, here is one truth about innovators: When they say they “innovated” a business model, they often mean that at the time, they did not have a clue about how to get their money back, and the business model simply did not exist. This is, perhaps, what happened here—the journal was a product of academic passion and did not aim to make money.

The *Journal of Medical Internet Research* was one of the first journals worldwide to use a new publishing model that empowers academics and enables end users like patients to access scientific information without going through a library. How early our contribution to this historic shift in scholarly publishing was can be illustrated by recalling some key dates in the short timeline of open access history: I conceived JMIR in 1998, with the first issue published in 1999, one year before early well-funded publishers like PLOS (incorporated as an advocacy organization in 2000 and a publisher in 2003) or BioMed Central (founded in 2000) entered the field. Because they were better funded and had marketing departments, they became the poster children for open access, but JMIR Publications certainly deserves credit for being an open access pioneer, as also evidenced by our role in cofounding the *Open Access Scholarly Publishers Association* (OASPA) [4], a nonprofit organization that promotes open access to health research and functions as a white list. Open access helps knowledge dissemination and uptake [5], but some overzealous publishers used questionable methods that threatened the credibility of open access. Concerns about the “black sheep” in the industry (later called “predatory” by Beall) arose about 10 years into our existence [6], and OASPA, created shortly after, was an important step to “whitelist” credible open access publishers.

Twenty years ago, the *Journal of Medical Internet Research* predicted that in the internet age, information needs to be open and free, and it went on to become extremely successful. It was soon named the leading eHealth journal in its category by Thomson Reuters (now Clarivate Analytics). Four journals, all published by JMIR Publications, are ranked within the top eight journals in the field, and two JMIR journals rank first (*Journal of Medical Internet Research*) and second (*JMIR mHealth and uHealth*) in this list. Although we warn people not to misuse the impact factor to assess researchers and institutions (there are better methods to rank the most impactful scientists [7]), publishing in a reputable journal remains critical for the career of an academic, and we would argue that all JMIR journals are reputable, irrespective of the impact factor. As one author put it, “publishing in JMIR is like getting on the honor roll.”

Soon after, the number of submissions started to increase exponentially, and we expanded our journal portfolio to cover a wide range of clinical specialties, to engage like-minded academics as editors-in-chief and to illustrate that “medical informatics” is not a “vertical” discipline but a horizontal theme that penetrates all areas of medicine—with JMIR as the journal brand that stands for innovation and digital progress in every single medical discipline. Today, JMIR Publications publishes over 2500 articles annually in nearly 30 journals.

It has not been an easy journey, and we faced opposition from expected and sometimes unexpected forces. Expected was pushback from scholarly publishing societies and large publishers, who are dependent on subscription revenues, and who did everything they could to delay the arrival and success of open access. Unexpected lack of support came from medical informatics societies, who have of course vested interests with their competing society-backed subscription/membership journals. But this may, perhaps, be a story for a different editorial.

Now is the time to celebrate our achievements in a positive light. We are celebrating our 20th anniversary with a special theme issue [8], which contains a remarkable mix of invited commentaries and original research, reflecting on the past and opening new perspectives for the near future. Of course, we are also celebrating JMIR itself while reflecting on our achievements and planning our direction for the future.

## The 20th Anniversary Special Issue

This special issue is truly remarkable, with exceptional articles and authors.

First, we provide a glimpse into the future of disruptive technologies, by publishing Elon Musk (yes, that one!) and his Neuralink team’s [9] paper about their groundbreaking *brain-machine interface (BMI)—*how to connect machines to the brain—for possible “read and write” access. The possibility to link ourselves directly to computers may address the bandwidth problem (especially for computer input), and we can even start dreaming about downloading apps that may directly program healthy habits into our brains or allow us to communicate with machines and each other wirelessly. The authors announced in a press conference earlier this year that they intend to start the first clinical trials as early as 2020. We are possibly on the brink of another revolution—the BMI revolution—the third revolution after the internet and mobile health (revolutions chronicled by 20 proud JMIR years).

The implications are equally fascinating and concerning, which is why Musk’s white paper is accompanied by four invited commentaries reflecting on the societal and scientific impact of this transformative technology [10-13]. They are scary, perhaps, because if we have not even figured out how to address the quality problem on the internet and in app stores, how can we ever hope to regulate and perform quality assessments for apps that may control our brains and are influenced by our brains, and work differently in each person—how do we assess their safety? There are many questions, so we are not concerned about running out of papers for the next 20 years.

Several papers in this issue also reflect on the rapid progress in *artificial intelligence* research in medicine [14-18]. Although these technologies have been around for decades, it seems that a tipping point has been reached, with these applications now becoming truly useful and mainstream in medicine. This is, in turn, partly fueled by the internet and the trillions of data points and textual information on it, giving machines the ability to understand the world better or, rather, to understand the world as *modelled on the internet* better. We have to stay mindful of the fact that the model of the world reflected on the internet is not a true representation of the world. Perhaps, herein lies a rarely discussed danger of artificial intelligence, in that machines will execute machine decisions based on a biased model of the world: Will *digital inequity* lead to or reinforce real-word inequity? This provides another angle on the importance of building capacity for digital health in resource-poor settings, as described by Walter Curioso in this issue [19].

Another section in this theme issue deals with the impact of the internet on patient empowerment. We are, for the first time, publishing the transcript of e-Patient Dave’s historic keynote at the Medicine 2.0 conference 10 years ago, which kicked off the #gmdd rallying cry and fueled the *participatory medicine* movement (JMIR Publications now also publishes the official journal of the Society for Participatory Medicine, the *Journal of Participatory Medicine*). Dave overcame cancer not least with the help of the internet and reminds us that patients need access to their medical data [20] - a goal that 10 years after his keynote is still not achieved [1].

We are also publishing the personal story of Andre Kushniruk, the editor-in-chief of *JMIR Human Factors*, who describes how the internet saved his life [21]. One morning, he woke up and had difficulties swallowing. A few weeks later, he started bleeding from his throat so profusely that he had to be rushed into the emergency department. The diagnosis was devastating—inoperable tongue cancer. The options he was given were chemotherapy and radiation, and these would be palliative only, with a slim chance of full recovery. However, with his wife, Elizabeth Borycki, who is also a professor at the School for Health Information in Victoria (and editor-in-chief of *JMIR Nursing*), the couple sprang into action, and after hours of searching on the internet, found a surgeon in the United States who specialized in surgically removing exactly the kind of cancer Andre had. The couple travelled to the United States for the surgery and today, Andre is free of cancer. “Freely available health information on the Internet saved his life,” says Elizabeth Borycki (who also contributed a paper to this theme issue on safety and error prevention in health care, which is not entirely unrelated to the power of informed patients to make health care better [22]).

It is stories like these that validate our belief in the power of the internet and digital technologies to “disintermediate” and not only disrupt the medical industry but also change the way researchers communicate their research results and patients retrieve information. However, open access to research information is not enough: Patients also need access to their personal health information, and the fact that we are not there yet is painfully illustrated in Dave DeBronkart’s latest article [1], which sometimes sounds bitter about the lack of progress and the forces resisting change. Disappointment and disillusionment are also reflected in the paper by Alex Jadad and his daughter [23]. These perspectives are sobering and refreshing at the same time, given the overly optimistic hype around digital health in other journals or news media, partly peddled by the “digital health” industry.

Speaking of hype and buzzwords, in this 20th year anniversary theme issue, Alireza Ahmadvand and colleagues [24] present a study on the increasing use of the *“digital health”* moniker in JMIR journals, a term I personally find problematic (not only because the word “digital” has another meaning in medicine, as anybody who has had a prostate exam can attest to that). Although we are not policing what terms people use in their papers, we gently try to dissuade authors from using these new buzzwords. We are also resisting the temptation to rename our flagship journal from *Journal of Medical Internet Research* to *JMIR Digital Health*, as buzzwords have a way of coming and going. More importantly, the term is not helpful, as it obfuscates the actual technologies used: For example, is it a Web-based intervention, a mobile intervention, or something connected to our brain? To write about a “digital intervention” is a little similar to writing about a “pharmacological intervention” without specifying the ingredients. Most importantly, having witnessed several terms come and go (“information and communication technologies [ICT],” “telemedicine,” “cybermedicine,” “eHealth,” “connected health,” “digital medicine,” etc), I now better understand the dynamics of why people create (and abandon) these new terms, and they are often related to money: venture capital funding for private companies or grants for funding research. Investors (of private companies) and peers (at funding agencies and journals) are often more impressed by something “new,” unencumbered by failures of the past, and eHealth is full of failures. But it is also full of success stories, and the reverse may happen as well: That something that works is forgotten and traded in for the next best shiny "digital health" thing. This sentiment is echoed in the paper by Jane C Willcox and colleagues [25], who remind us that textmessaging is still one of the most effective health interventions, but funding bodies tend to turn down proposals with technologies that are not new and sexy enough. Thus, my biggest concern with the new "digital health" hype and -more broadly- with constantly rebranding something that has been around for decades, is that it affects our ability to learn from failures or successes of the past. To abandon a term like electronic health/electronic medicine in favor of “digital health” or “digital medicine,” only to appear cutting edge and prevent people from googling or researching past failures and successes associated with the field is dishonest and antiscientific, and we will not stand for it. Although the word “internet” in our flagship journal title may no longer adequately and exhaustively describe what we publish, the internet remains the fuel for innovation and the infrastructure that enables global communication, and we will continue publishing cutting-edge research no matter what the buzzword *du jour* is.

## Open Access is Knowledge, and Knowledge is Power

**Figure 1:**
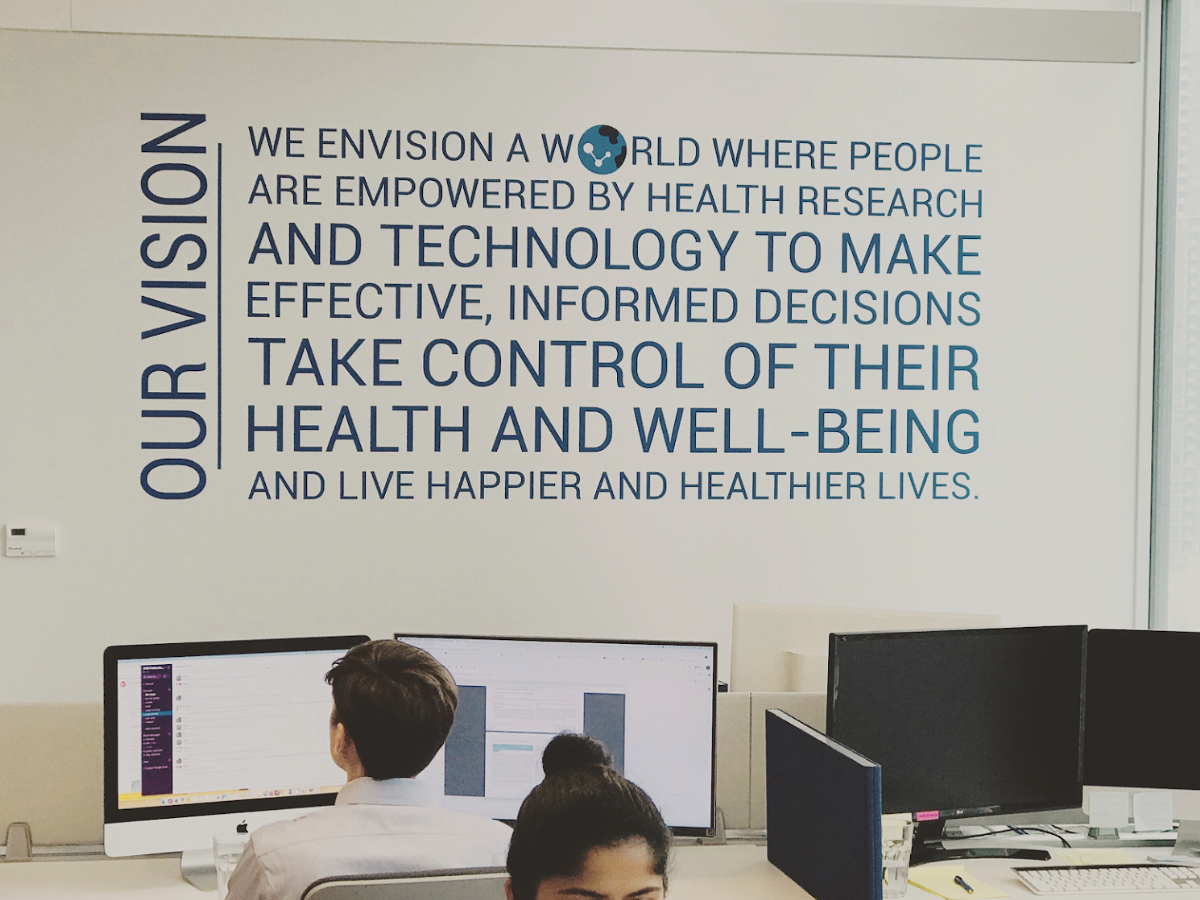
Vision of JMIR Publications at the wall of the editorial office.

The stories of e-Patient Dave [20] or Andre Kushniruk [21] exemplify what I tried to change 20 years ago, why the journal was created in the first place, and why I decided to make it open access. JMIR Publications is not only a business, but also a social enterprise: We try to instigate social change, as evidenced by our vision statement, which is engraved on the wall of our head office in Toronto, Canada (Figure 1):

We envision a world where people are empowered by health research and technology to make effective, informed decisions, take control of their health and well-being, and live happier and healthier lives.

John Torous, our brilliant Editor-in-chief of *JMIR Mental Health*, writes wonderfully about our impact beyond the impact factor [26], but what I am most proud of is our role in advancing two important social movements of our time: Open Access (to free general medical knowledge from being behind paywalls) [4,5], and participatory medicine (which includes and necessitates freeing personal health information from the vaults of institutional medicine) [20]. As publisher, we are helping likeminded leaders in the health technology space collaborate and disseminate their ideas and research results, facilitating progress and change. Some of our readers indeed recognize our role as social change agent: "JMIR is an interesting and socially engaged enterprise worthed to publish with" writes Alberto J. Revolware in a Google review [27]. Others call us "a very forward-thinking publication (..). A true beacon of light for the digital health industry." (Jessica Shull) [27]. Most authors also appreciate the innovations we made to streamline the review and publishing process, with industry-leading turnaround times and impact: "JMIR has completely optimized the process of reviewing and publishing manuscripts. Other journals should follow suit." writes Jereme Wilroy [27].

To achieve our vision, we not only publish research papers, but also create and apply additional tools that connect vetted, quality research outputs in novel, effective, and timely ways for those who need it, and that includes patients. Therefore, in addition to its 30 peer-reviewed journals, JMIR Publications maintains a blog site for end users and plans to translate scientific research into consumer-understandable, actionable information (watch our forthcoming product announcements). Organizing and supporting medical and scientific conferences; developing new software, databases, and resources for researchers and patients alike; and even angel investment in small digital health and digital science companies are all part of our toolbox toward that goal and go beyond publishing.

Academic societies take notice of these efforts and increasingly move their journals to JMIR Publications. When the nonprofit *Society for Participatory Medicine* was looking for a new home for its *Journal of Participatory Medicine*, JMIR Publications was a natural fit, and the society moved the journal to JMIR Publications. The movement in which networked patients shift from being mere passengers to responsible drivers of their health and where providers encourage and value patients as full partners, goes well beyond digital medicine, but information technology is a catalyst to enable that paradigm shift.

We also forge partnerships with research institutions and libraries; for example, owing to a recent deal with the California Digital Library at the University of California (UC), the article processing fees for authors from all 10 UC campuses throughout California are subsidized or fully covered through this agreement. We will continue to try to make similar institutional or even national agreements, but we will also need the help of librarians, faculty, and our readers to advocate such deals at their institutions or national libraries.

## The Future of Publishing: Our Innovations for 2020 and the Next 20 Years

We are not done innovating, and we are very excited to announce the launch of the new JMIRx journal series, which expands our scope beyond electronic health to all areas of medicine, and even biology and psychology, and provides an entirely new publishing experience for authors.

**Figure 2:**
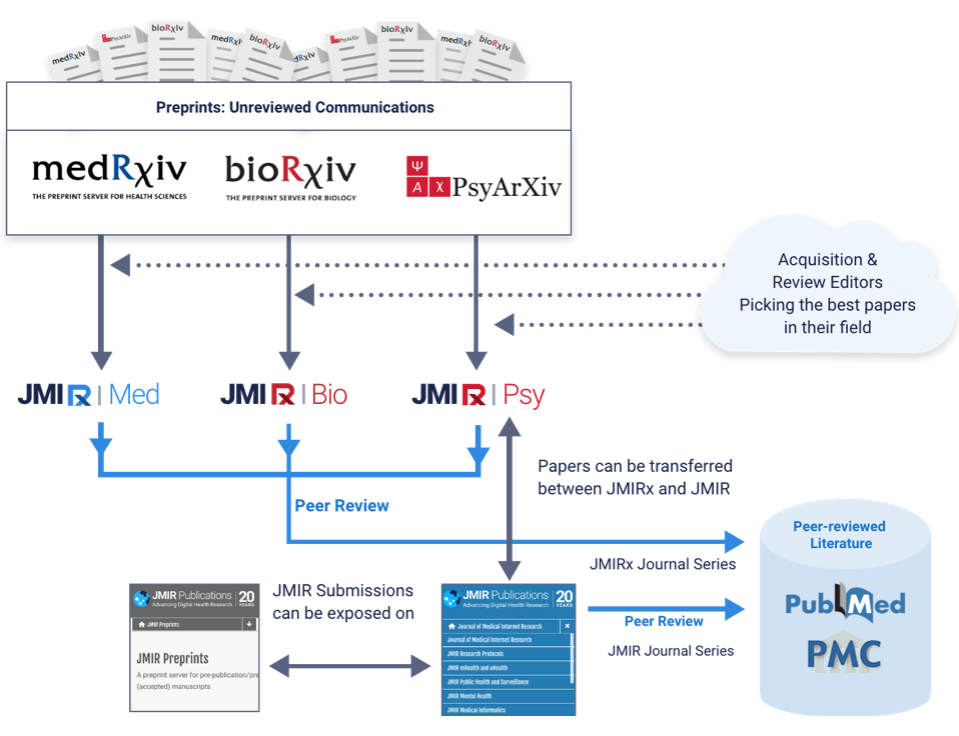
JMIRx, a new series of superjournals (overlay journals) offering peer review and publication services for preprints.

JMIRx is a new journal series and new type of journal, which we call “superjournals” (others may call them “overlay” journals; Figure 2). Superjournals are operating “on top” of preprint servers and offer peer review and copyediting/archiving/indexing services; as such, they do everything a “normal” journal does, except that authors no longer have to submit their manuscript to a journal. Instead, our acquisition and review editors find the papers they want to publish and extend conditional offers of publication to interesting articles published in preprint servers as well as solicit reviews and commentaries. In addition to the “editorial prospecting” workflow, authors can self-nominate their existing preprints for publication (which is equivalent to a traditional journal submission) without going through another submission process. If superjournals ask for revisions, these revisions are also uploaded to the preprint server.

We see this model, which includes radical openness during the entire manuscript preparation and revision process, as the future of publishing, as predicted 20 years ago in the year 2000, when I reflected on the power of preprint servers and the blurring boundaries between what constitutes a “publication” (rather than the traditional dichotomy of “published” versus “unpublished” articles I posited that the internet age calls for a distinction between “type 1” [informal] and “type 2” [version of record, peer-reviewed] publications):

Researchers could submit type-1 electronic papers [preprints] to preprint servers for discussion and peer-review, and journal editors and publishers would pick and bid for the best papers they want to see as “type-2 papers” [version of record] in their journal. [28]

This is what I still believe may be the future of publishing, and JMIRx may illustrate this. Combined with other innovations, such as our *JMIR Research Protocols* journal, which increases accountability and transparency in research through registered protocols and registered reports, we are confident that once again, we are at the at cutting edge of an open-science revolution, and we look forward to take that journey in the next 20 years with our growing base of readers, editors and reviewers.

## Abbreviations

BMI: brain-machine interface
eHealth: electronic health
ICT: information and communication technologies
JMIR: Journal of Medical Internet Research
OASPA: Open Access Scholarly Publishers Association
UC: University of California
